# Effects of Soluble and Insoluble Fibre on Glycolipid Metabolism and Gut Microbiota in High-Fat-Diet-Induced Obese Mice

**DOI:** 10.3390/nu16223822

**Published:** 2024-11-07

**Authors:** Han Ren, Sihao Dong, Li Li, Wei Zhao

**Affiliations:** 1 State Key Laboratory of Food Science and Resources, Jiangnan University, Wuxi 214122, China; 6220112062@stu.jiangnan.edu.cn (H.R.); lili.zz@jiangnan.edu.cn (L.L.); 2 School of Food Science and Technology, Jiangnan University, Wuxi 214122, China; 3 Zhejiang Qianhuxue Wine Industry Co., Ltd., Shaoxing 325000, China; dongsihao@vegehero.cn

**Keywords:** barley leaf, insoluble dietary fibre, soluble dietary fibre, obesity, glycolipid metabolism, gut microbiota

## Abstract

Background: Dietary fibre can alleviate or reduce the risk of obesity and obesity-induced abnormalities in glycolipid metabolism. However, the effects of different types of dietary fibre or their combinations on obesity remain unclear. Here, we explored the effects of different ratios of inulin soluble dietary fibre (ISDF) and barley leaf insoluble dietary fibre (BLIDF) on the body weight, glycolipid metabolism and gut microbiota of obese mice. Methods: Seven experimental groups were treated with different combinations of soluble and insoluble fibre, comprising HFD (high-fat diet without dietary fibre), BLIDF, ISDF, I3S1DF (insoluble/soluble = 3:1), I2S2DF (insoluble/soluble = 1:1), I1S3DF (insoluble/soluble = 1:3) and MIX (inulin, BLIDF and matcha powder fibre; insoluble/soluble = 3.6:1) groups. Results: Our results showed that the BLIDF, ISDF and MIX treatments decreased the body weight gain of the HFD mice significantly after eight-week interventions. All the fibre intervention groups except the MIX group displayed lower fasting blood glucose and glycosylated serum protein levels than the HFD group. BLIDF, ISDF, I3S1DF and I2S2DF improved the glucose tolerance of the mice. Moreover, none of the dietary fibre interventions affected the liver lipid metabolism, while I3S1DF and I1S3DF improved the abnormal serum lipid metabolism. BLIDF, ISDF, I3S1DF and I2S2DF reduced the serum IL-6 levels, and BLIDF and I1S3DF increased SOD activity significantly. Additionally, all the dietary fibre interventions decreased the Firmicutes to Bacteroidota (F/B) ratio and increased the abundance of beneficial gut microbes differently. Conclusions: In short, our results suggest that different ratios of soluble and insoluble dietary fibre have unique impacts on mice body weight, glycolipid metabolism, inflammation and gut microbiota. The ratio of soluble to insoluble dietary fibre intake should be considered for specific health goals in the future.

## 1. Introduction

Over the past few years, the world has encountered significant health challenges. According to a recent study in The Lancet, the global obesity population has surpassed one billion people [1]. The primary cause of obesity is an imbalance between energy intake and expenditure. Obesity can lead to increased lipid levels, liver function abnormalities, oxidative stress and various chronic metabolic diseases. Additionally, obesity can induce negative psychological states, resulting in more serious health consequences [2]. The prolonged adoption of unhealthy lifestyles, characterized by diets high in fat and sugar, disrupts the physiological regulation of glucose and lipid metabolism within the body, leading to the induction of chronic low-grade inflammation. This inflammatory state subsequently elevates the risk factors associated with obesity, cardiovascular diseases, diabetes mellitus and other chronic health conditions.

Numerous epidemiological and intervention studies have demonstrated that high-fibre diets can improve abnormalities in glycolipid metabolism and reduce the risk of obesity [3]. Different types of dietary fibre (from different sources, soluble or insoluble dietary fibre) possess unique physicochemical properties, display distinct functions and different anti-obesity effects and exert their beneficial effects via different pathways. Some studies have shown that the consumption of a diet high in soluble fibre not only reduced body weight gain and the excessive accumulation of white fat tissue, but also lowered total cholesterol and low-density lipoprotein cholesterol [4,5], while Peng et al. found that insoluble fibre had the ability to ameliorate the changes in gut microbiota induced by a high-fat diet [6]. Soluble and insoluble dietary fibres have different properties and health benefits. Our prior research has demonstrated that the administration of soluble dietary fibre derived solely from flaxseed cake, or a combination of both soluble and insoluble dietary fibres from flaxseed cake, resulted in a reduction in fat accumulation, an improvement in the serum lipid profile and an elevation in the basal metabolic rate in mice, while the consumption of insoluble dietary fibre isolated from flaxseed cake alone led to an enhancement in the liver lipid profile, an augmentation of the basal metabolic rate and an increase in the levels of short-chain fatty acids within the guts of the mice [7].

Research on the human microbiome has revealed the critical role of bacteria in human health and disease. The gut microbiota fulfils a crucial role in sustaining the health of the host through the provision of energy, nutritional substrates and immune protection [8]. It also regulates fat absorption, transport, storage and metabolism [9]. By modulating the gut microbiome with beneficial dietary interventions, the integrity of the gut barrier can be improved, ameliorating chronic inflammation and metabolic disorders linked to obesity. Several studies have shown that individuals with obesity, both in animal models and humans, exhibit an elevated proportion of *Firmicutes* relative to *Bacteroidota*, compared to those maintaining a normal weight. This finding implicates the *Firmicutes*-to-*Bacteroidota* ratio as a pivotal biomarker [10]. Dietary fibres can affect host gut microbiota composition significantly and may regulate obesity via gut microbiota [11].

Barley, characterized by its high protein, high fibre, high vitamin, low fat and low sugar levels, aligns well with modern nutritional requirements. Researches have demonstrated that barley fibre exhibited the capacity to reduce blood glucose and lipid concentrations, foster intestinal health and augment immune function [12,13]. Our study aims to investigate the effects and mechanisms of different ratios of soluble dietary fibres (inulin) and insoluble dietary fibres (from barley leaf) on body weight and glycolipid metabolism in obese mice. By measuring body weight, serum glycolipid metabolism-related indexes, energy metabolism and gut microbiota in mice, we seek to determine the optimal ratio of soluble and insoluble dietary fibres intake for body weight control and the homeostasis of glucolipid metabolism. The results will provide new strategies for effective treatments of obesity, hyperglycemia and hyperlipidemia.

## 2. Materials and Methods

### 2.1. Analysis of the Nutrient Composition of Barley Leaf Powder

Barley leaf powder (BLP), purchased from Shaoxing Imperial Tea Village Tea Industry Co., Ltd. (Shaoxing, China), was used in our study and its nutrient composition was first analyzed. The content of protein, fat, moisture and ash in the barley leaf powder was determined according to GB 5009.5-2016 [14], GB 5009.6-2016 [15], GB 5009.3-2016 [16] and GB 5009.4-2016 [17] standards, respectively. The dietary fibre content was measured using Total Dietary Fibre (TDF) kits purchased from Megazyme (Shanghai, China), while the starch content was determined using Starch Content Detection kits purchased from Meryer (Suzhou, China).

### 2.2. Extraction and Determination of Dietary Fibres from Barley Leaf Powder

In this experiment, dietary fibre from barley leaf powder was extracted by enzymatic digestion using the Zhang’s method with minor modification [18]. First, 10 g of barley leaf powder was dissolved in deionized water at a ratio of 1:20 (*w*/*v*), and then the solution was mixed with α-amylase (0.5% *v*/*v*) and kept at 95 °C for 1 h. After cooling the solution to 60 °C, the pH was adjusted to 9.5 ± 0.2, and alkaline protease (0.6% *v*/*v*) was added and incubated at 60 °C for 3 h. Subsequently, enzyme inactivation was performed at 100 °C for 10 min post-digestion. The solution was centrifuged with 7100× *g* at 4 °C for 10 min to separate into supernatant A and precipitate B. The supernatant A was mixed with four times its volume of 95% ethanol (*v*/*v*), which was precipitated for 12 h. The resulting precipitate was vacuum-dried to obtain barley leaf soluble dietary fibre (BLSDF). The precipitate B was washed with distilled water first, then with 95% ethanol and finally with distilled water. The collected precipitate was vacuum-dried to obtain barley leaf insoluble dietary fibre (BLIDF). The BLSDF and BLIDF were crushed and sieved through a 60-mesh sieve. The purity of the BLIDF was 90.97%.

### 2.3. Scanning Electron Microscopy (SEM) Analysis

The BLIDF powder obtained from Section 2.2 was freeze-dried for 48 h to remove water. Subsequently, under low-pressure conditions, the dried BLIDF powder samples were placed on the sample stage and then underwent a sputter-coating process with gold. Following this preparatory step, scanning was performed using a cold field emission scanning electron microscope (FEI Inc., Hillsboro, OR, USA) at an accelerating voltage of 20 kV. The micromorphological structure of BLIDF was discerned through precise adjustments of the magnification and zoom settings.

### 2.4. Animal Experiments

Seventy 12-week-old SPF grade C57BL/6JGpt DIO male mice (obese mice; 35 g ± 2 g) were purchased from Jiangsu Jicui Pharmachem Bio-technology Co., Ltd. (Nanjing, China). All the animal experimental procedures were approved by the Experimental Animal Management and Animal Welfare Ethics Committee of Jiangnan University (JN.No.20231115c0700330[527]) and were conducted in accordance with the fundamental guidelines for the Care and Use of Laboratory Animals. The mice were housed in a specific pathogen-free environment at the Jiangnan University Laboratory Animal Center (SYXK(SU)2021-0056, Wuxi, China) under the following conditions: temperature 22 ± 2 °C, humidity 55 ± 5% and a strict 12 h light/dark cycle. All the mice had free access to food and water during the rearing period. After one week of acclimatization, the mice were randomly divided into seven groups (*n* = 10 per group): the group fed with a high-fat diet without dietary fibre (HFD group), the groups fed with a high-fat diet containing 5% BLIDF (BLIDF group), containing 5% inulin soluble dietary fibre (ISDF) (ISDF group), containing 3.75% BLIDF and 1.25% ISDF (I3S1DF group), containing 2.5% BLIDF and 2.5% ISDF (I2S2DF group) or containing 1.25% BLIDF and 3.75% ISDF (I1S3DF group) and the group fed with a high-fat diet containing 5% mixed dietary fibre including inulin, BLIDF and matcha powder fibre (the soluble to insoluble dietary fibre ratio was approximately 3.6:1; MIX group). During the experiment, the body weight and fasting blood glucose (FBG) of the mice were measured weekly. Additionally, 24 h food and water intake were recorded once a week.

An oral glucose tolerance test (OGTT) was performed during the last week of the experiment. The mice were fasted for 14 h and then were gavaged with sterile glucose solution (2 g/kg body weight). The blood glucose levels were measured at 0, 30, 60, 90 and 120 min by collecting blood from the tail tip using blood collection needles (0 min represents the fasting glucose level of the mice before gavage). OGTT plots were drawn, and the area under the curve (AUC) was calculated.

In the final week of the experiment, the 24 h energy metabolism of the mice was monitored using the Comprehensive Laboratory Animal Monitoring System (CLAMS) (Columbus Instruments, Columbus, OH, USA). The oxygen consumption (VO_2_), carbon dioxide production (VCO_2_) and thermal energy output were evaluated in order to gain insight into the physiological processes occurring within the body. The respiratory exchange ratio (RER) was calculated as VCO_2_/VO_2_. Fresh mouse faeces were collected in sterile centrifuge tubes before the mice were sacrificed and frozen at −80 °C. One day before the end of the experiment, the mice were fasted for 12 h (with free access to sterile water). After the experiment, the mice were weighed. Then, they were anesthetized with 2% isoflurane. Subsequently, 0.6–1 mL of blood was collected by enucleation (eyeball removal), and the mice were euthanized by cervical dislocation (neck breaking). The collected blood samples were centrifuged with 1309× *g* at 4 °C for 10 min, and the supernatant was collected and stored at −80 °C. The brown adipose tissue (BAT), epididymal white adipose tissue (eWAT) and inguinal subcutaneous adipose tissue (iWAT) of the mice were separated, rinsed with saline, weighed and then frozen in liquid nitrogen. Liver caudate tissues were collected, with a portion of them being fixed in 4% paraformaldehyde solution for the purpose of histological staining; the rest were snap-frozen in liquid nitrogen and stored at −80 °C.

### 2.5. Detection of Serum Biochemical Indexes

The total cholesterol (TC), triglyceride (TG), high-density lipoprotein cholesterol (HDL-C), low-density lipoprotein cholesterol (LDL-C), total bile acid (TBA) and uric acid (UA) levels were measured using a fully automated biochemistry analyzer (Mindrayn BS-480, Shenzhen, China). The glucagon-like peptide-1 (GLP-1), interleukin-6 (IL-6) and interleukin-10 (IL-10) levels in the serum were analyzed using Mouse ELISA Kits (ml201801, ml063159, ml037873) purchased from Shanghai Enzyme-linked Biotechnology (Shanghai, China). The insulin level was measured using a Mouse Insulin ELISA Kit (Ultrasensitive) purchased from Beyotime Biotechnology (Shanghai, China). The glycosylated serum protein (GSP) content was detected by using a glycosylated serum protein assay kit purchased from Nanjing Jiancheng Bioengineering Institute (Nanjing, China).

### 2.6. Analysis of Organ Indexes

The BAT, eWAT and iWAT were rinsed with saline. The surface moisture was then absorbed by filter paper, after which the tissues were weighed in order to ascertain their respective indexes. The calculation of each tissue’s index was performed using a specific formula:

Organ index (%) = tissue weight/body weight × 100%.(1)

### 2.7. Detection of Liver Biochemical Indexes

An appropriate amount of liver tissue was taken and mixed with ice-cold saline. The tissue was then homogenized at a low temperature using a tissue grinder and centrifuged at 1327× *g* for 10 min. The supernatant was collected for further analysis. The TC and TG contents in the mouse liver were determined using assay kits (A111-1-1, A110-1-1) from Nanjing Jiancheng Bioengineering Institute (Nanjing, China). The malondialdehyde (MDA) content was measured using the mouse malondialchehyche (MDA) ELISA Kit (Shanghai Enzyme-linked Biotechnology, Shanghai, China). The superoxide dismutase (SOD) activity was analyzed using an assay kit (BL1748B) purchased from Biosharp life sciences (Hefei, China).

### 2.8. Histological Analysis

The liver caudate tissues of the mice were stabilized in 4% paraformaldehyde for a duration of 48 h. Following this, the tissues were embedded, sliced and then stained with hematoxylin–eosin (HE). Subsequently, the slices were dehydrated with gradient alcohol and sealed with neutral gum. Finally, the stained sections were observed under a microscope (Nikon Eclipse E100, Tokyo, Japan), and the images were recorded and analyzed.

### 2.9. Gut Microbiota Analysis

The principal objective of this experiment was to elucidate the microbial composition within mouse faeces. To achieve this, 16S rRNA amplicon sequencing was performed. The extraction of DNA from the mice faeces was conducted using a FastPure Stool DNA Isolation Kit (Magnetic bead) (MJYH, Shanghai, China). The V3-V4 hypervariable region of the extracted DNA samples was amplified using the universal primers 338F and 806R. PCR amplicons were extracted and purified by an AxyPrep DNA Gel Extraction Kit (Axygen Biosciences, Union City, CA, USA). Subsequently, the purified amplicons were paired-end sequenced on the Illumina Miseq PE300 platform (Illumina, San Diego, CA, USA). The operational taxonomic units (OTUs) were clustered using UPARSE software (version 7.1) with 97% sequence similarity. The alpha diversity index was calculated by the Mothur software (version 1.48.2). Principal coordinate analysis (PCoA) based on the Bray–Curtis distance was performed to observe the differences in microbial community structure between the samples. Furthermore, linear discriminant analysis effect size (LEfSe, LDA > 2, *p* < 0.05) was applied to identify bacterial genera with significant differences in relative abundance among groups.

### 2.10. Statistical Analysis 

Data analysis was performed using Origin 2018 (OriginLab, Northampton, MA, USA) and IBM SPSS Statistics 22 (IBM, Armonk, NY, USA). All the data are expressed as mean ± standard deviation (SD) calculated based on a minimum of three replicates. Statistical significance was analyzed using one-way analysis of variance (ANOVA) with Tukey’s test and Duncan’s test for multiple comparisons. A *p*-value less than 0.05 (*p* < 0.05) is considered significant in all analyses, which is the conventional threshold for determining statistical significance in the field of scientific research.

## 3. Results and Discussion

### 3.1. The Dietary Fibre in Barley Leaf Powder 

**Figure 1:**
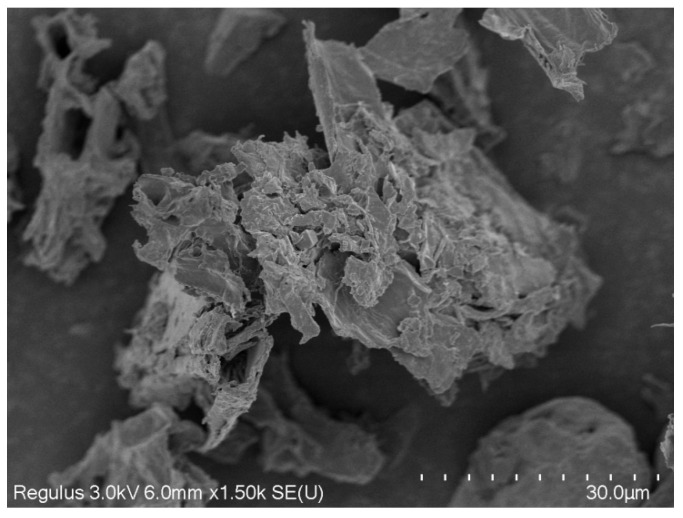
SEM analysis of BLIDF at 1500× magnification.

The dietary fibre and other nutrient composition in barley leaf powder are first measured in our study by the national standard methods. As shown in Table 1, the barley leaf powder contained a high percentage of dietary fibre, with insoluble dietary fibre accounting for 55.46% and soluble dietary fibre accounting for 0.89%. This indicates that most of the dietary fibre in barley leaf powder is insoluble. Scanning electron microscopy (SEM) observations revealed that the barley leaf insoluble dietary fibre (BLIDF) had a wrinkled surface with voids, large and irregular particles and a large specific surface area, which enhanced its ability to absorb water and oil (Figure 1). The purity of the BLIDF extracted by the enzymatic digestion method in our study was 90.97%, which was used for subsequent animal experiments.

### 3.2. Effects of Soluble and Insoluble Dietary Fibre Ratios on Body Weight of Obese Mice

**Figure 2:**
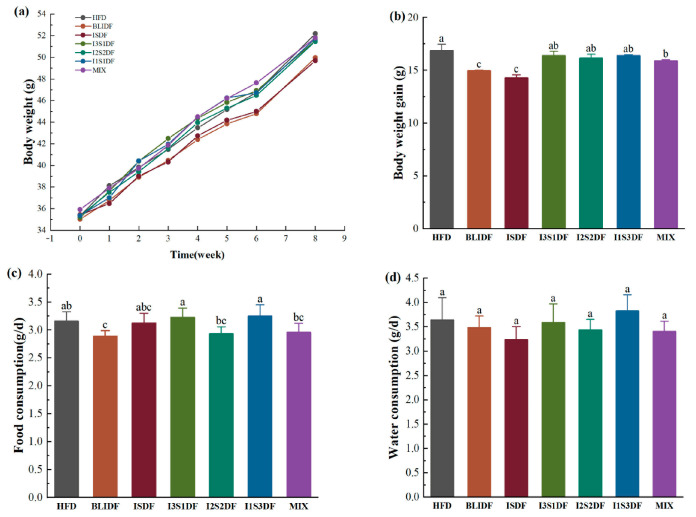
Effects of soluble and insoluble dietary fibre ratio on the body weight of obese mice. (**a**) Body weight. (**b**) Body weight gain after an eight-week dietary fibre intervention. (**c**) Food consumption. (**d**) Water consumption. Data are presented as means ± SD (*n* = 10). Differences between each group corresponding to different letters are statistically significant (*p* < 0.05).

Soluble and insoluble dietary fibre have distinct functions. Here, we initially investigated the impact of various soluble to insoluble dietary fibre ratios on the body weight of high-fat-diet-induced obese mice. The soluble dietary fibre used was inulin, and the insoluble dietary fibre was the barley leaf insoluble dietary fibre (BLIDF) extracted above. The obese mice (HFD mice) were fed a high-fat diet without dietary fibre, or were fed with 5% BLIDF, 5% inulin soluble dietary fibre (ISDF) or 5% dietary fibre with soluble to insoluble dietary fibre ratios of 1:3, 1:1 and 3:1. Additionally, one group of the mice received a mixture of dietary fibre (including inulin, BLIDF and matcha powder fibre) with a soluble to insoluble dietary fibre ratio of approximately 3.6:1. As shown in Figure 2a, the body weight of the mice in the BLIDF and ISDF groups increased more slowly compared to the other groups. Moreover, after an eight-week intervention period, the BLIDF, ISDF and MIX treatments significantly attenuated the body weight gain of the mice compared to the HFD group (Figure 2b). This is consistent with prior research that eating food rich in soluble fibre can reduce weight gain [4], which indicates that BLIDF, ISDF and MIX dietary fibre intervention can significantly reduce the body weight of obese mice. During this period, the BLIDF group demonstrated a reduction in food consumption compared to the HFD group. There was no significant difference in food consumption among the other groups (Figure 2c). BLIDF had a wrinkled surface with gaps, enhancing its ability to absorb water and oil. This may make the mice feel fuller, leading to reduced food intake. Additionally, no statistically significant difference in the water intake of the mice was observed among the groups (Figure 2d), indicating that the fibre intervention did not affect water requirements of the mice.

### 3.3. Effects of Soluble and Insoluble Dietary Fibre Ratios on Glucose Homeostasis of Obese Mice 

**Figure 3:**
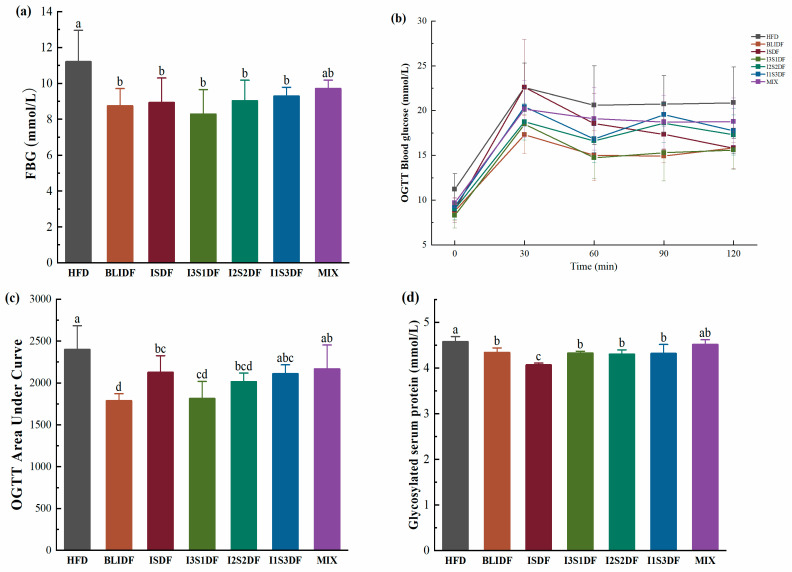
Effects of soluble and insoluble dietary fibre ratio on the glucose homeostasis of obese mice. (**a**) Fasting blood glucose. (**b**,**c**) OGTT (**b**) and AUC of OGTT (**c**) in mice with different dietary fibre interventions. (**d**) Glycosylated serum protein levels in serum. Data are presented as means ± SD (*n* = 10). Differences between each group corresponding to different letters are statistically significant (*p* < 0.05).

Obesity is accompanied by an imbalance in glucose metabolism. Thus, the impact of various soluble to insoluble dietary fibre ratios on glucose homeostasis and insulin response was evaluated. Fasting blood glucose (FBG) reflects pancreatic β-cell function and generally indicates basal insulin secretion. After 7 weeks’ fibre intervention, the FBG levels of the mice within each experimental group were assessed. The corresponding results are presented in Figure 3a. All the fibre interventions, except for the mixed fibre intervention (the MIX group), significantly reduced the FBG level of the obese mice compared to that of the HFD mice without fibre intervention. 

The OGTT serves as a diagnostic instrument for observing alterations in blood glucose concentrations. This test involves administering a precise quantity of glucose to the subjects, which in turn allows for the assessment of the insulin secretory function of pancreatic islet β-cells and the body’s capacity to regulate glucose metabolism. Thus, the OGTT was then conducted. Our results showed that the blood glucose levels in each group peaked 30 min after glucose intake and gradually decreased thereafter (Figure 3b). The peak blood glucose levels in the HFD and ISDF group were significantly higher than those in the other groups. Upon comparing the AUC of the OGTT among the various groups, a significant decrease was observed in the BLIDF, ISDF, I3S1DF and I2S2DF groups when compared to the HFD group. These results indicated that the BLIDF, ISDF, I3S1DF and I2S2DF intervention may enhance glucose tolerance in mice, as illustrated in Figure 3c. Specially, BLIDF had the most pronounced effect in reducing the peak blood glucose level and AUC value. It seems that more insoluble dietary fibre can improve the glucose tolerance of obese mice more efficiently.

Glycosylated serum protein (GSP) can reflect the average blood sugar levels in the most recent 2–3 weeks [19]. All the dietary fibre intervention groups except the MIX group had significantly lower GSP content than the HFD group (Figure 3d). 

**Figure 4:**
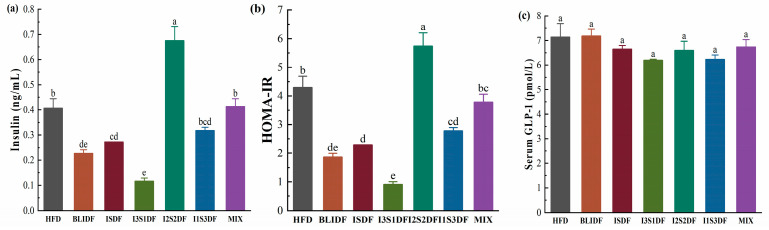
Effects of soluble and insoluble dietary fibre ratios on mice insulin response. (**a**) Insulin. (**b**) HOMA-IR. (**c**) GLP-1. Data are presented as means ± SD (*n* = 6). Differences between each group corresponding to different letters are statistically significant (*p* < 0.05).

Insulin promotes the uptake and utilization of glucose by body cells and inhibits glycogen breakdown, thus reducing blood glucose levels. The serum insulin levels of the mice in each group were determined, and HOMA-IR was calculated. As shown in Figure 4a, the insulin level of the obese mice significantly decreased in the BLIDF, ISDF and I3S1DF groups, but significantly increased in the I2S2DF group compared to the HFD group. Moreover, the BLIDF, ISDF, I3S1DF and I1S3DF groups showed a significant decrease in insulin resistance compared to the HFD group (Figure 4b). Insulin resistance, considered a potential pathological mechanism of many metabolic syndromes and cardiovascular diseases (including obesity), can decrease insulin efficiency. GLP-1 is an incretin secreted by intestinal endocrine L cells, stimulating insulin secretion to reduce blood glucose levels and promoting the proliferation and differentiation of β cells [20]. As illustrated in Figure 4c, no statistically significant difference in GLP-1 levels was observed among the various groups, indicating that dietary fibre interventions did not significantly impact the regulation of this incretin hormone.

### 3.4. Effects of Soluble and Insoluble Dietary Fibre Ratios on Fat Accumulation and Lipid Profiles of Obese Mice

**Figure 5:**
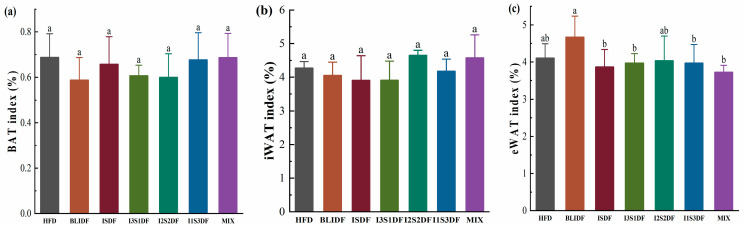
Effects of soluble/insoluble dietary fibre ratio on organ index in mice. (**a**) BAT index. (**b**) iWAT index. (**c**) eWAT index. Data are presented as means ± SD (*n* = 8). Differences between each group corresponding to different letters are statistically significant (*p* < 0.05).

**Figure 6:**
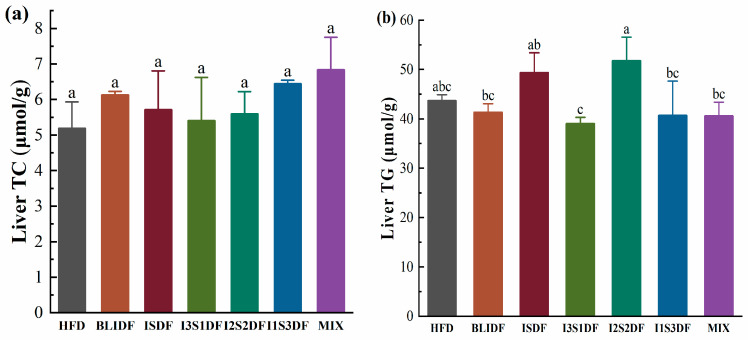
Effects of soluble/insoluble dietary fibre ratio on liver lipid metabolism in mice. (**a**) TC. (**b**) TG. Data are presented as means ± SD (*n* = 6). Differences between each group corresponding to different letters are statistically significant (*p* < 0.05).

Obesity is especially associated with an imbalance in lipid metabolism. Here, we investigated the soluble and insoluble dietary fibre ratios on fat accumulation and lipid profiles in the liver and serum of obese mice. As shown in Figure 5, none of the dietary fibre interventions changed the BAT, iWAT and eWAT index compared to that of the mice in the HFD group. Further, we found that there were no significant differences in the TC and TG content in the liver of the mice between any two groups (Figure 6). It seems that the dietary fibre interventions did not affect the liver’s fat accumulation and lipid metabolism.

**Figure 7:**
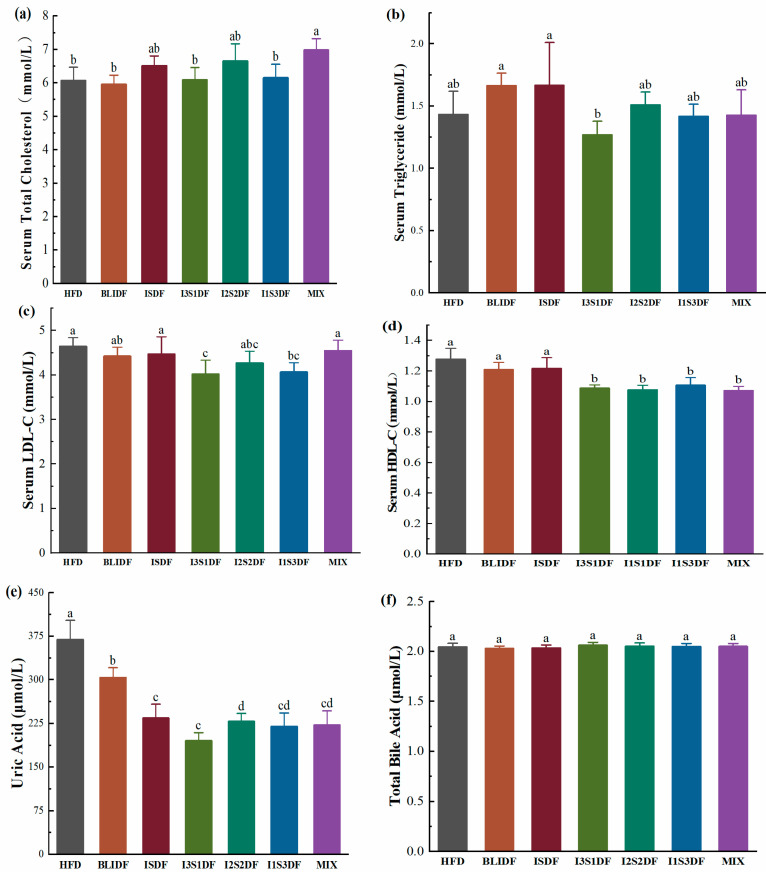
Effects of soluble and insoluble dietary fibre ratios on serum lipid profiles of obese mice. (**a**) TC. (**b**) TG. (**c**) LDL-C. (**d**) HDL-C. (**e**) UA. (**f**) TBA. Data are presented as means ± SD (*n* = 6). Differences between each group corresponding to different letters are statistically significant (*p* < 0.05).

Regarding the serum lipid profile analysis, the results showed that, as illustrated in Figure 7a, the MIX group had a higher TC level compared to the HFD group, while the I3S1DF and I1S3DF groups exhibited a significant reduction in serum LDL-C levels compared to the HFD group (Figure 7c). Moreover, the I3S1DF, I2S2DF, I1S3DF and MIX groups showed a significant decrease in serum HDL-C levels compared to the HFD group. In contrast, the BLIDF or ISDF intervention alone did not significantly affect the serum HDL-C levels in the mice. Compared to the HFD group, none of the dietary fibre interventions altered the serum TG (Figure 7b). Elevated concentrations of TC are correlated with the advancement of coronary heart disease and the development of atherosclerosis. LDL-C can transport cholesterol into peripheral tissues, and elevated cholesterol levels can contribute to atherosclerosis [21]. It can be seen that I3S1DF and I1S3DF can alleviate the abnormal serum lipid metabolism. Further, all the fibre interventions reduced UA levels (Figure 7e), and no significant difference in TBA content between the groups was observed (Figure 7f). UA may contribute to the onset of conditions such as hyperuricemia, gout and nephritis [22]. Our results showed that dietary fibres have the capacity to lower UA levels, which may reduce the incidence of this series of diseases.

### 3.5. Effects of Soluble and Insoluble Dietary Fibre Ratios on Energy Expenditure and RER of Obese Mice

**Figure 8:**
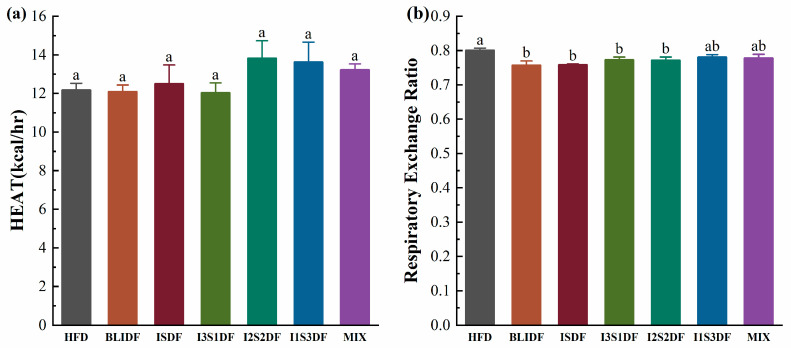
Effects of soluble and insoluble dietary fibre ratios on energy metabolism of obese mice. (**a**) HEAT. (**b**) RER. Data are presented as means ± SD (*n* = 4). Differences between each group corresponding to different letters are statistically significant (*p* < 0.05).

Obesity arises due to an imbalance between energy intake and expenditure, specifically characterized by excessive energy intake relative to insufficient energy expenditure. So, we examined the energy expenditure and RER of the obese mice in each group. Our results indicated no statistically significant difference in energy expenditure among the groups (Figure 8a), while the RER values in all the groups were less than 1, and the RER values were significantly reduced in the BLIDF, ISDF, I3S1DF and I2S2DF groups compared to the HFD group (Figure 8b). An RER of 1 indicates aerobic respiration using glucose as the sole substrate, while an RER of less than 1 indicates aerobic respiration using fat as the substrate [23]. The results suggest that the BLIDF, ISDF, I3S1DF and I2S2DF dietary fibre interventions could increase the capacity of the mice to utilize fat for energy, contributing to weight reduction.

### 3.6. Effects of Soluble and Insoluble Dietary Fibre Ratios on Inflammation Levels and Antioxidant Ability in Obese Mice

**Figure 9:**
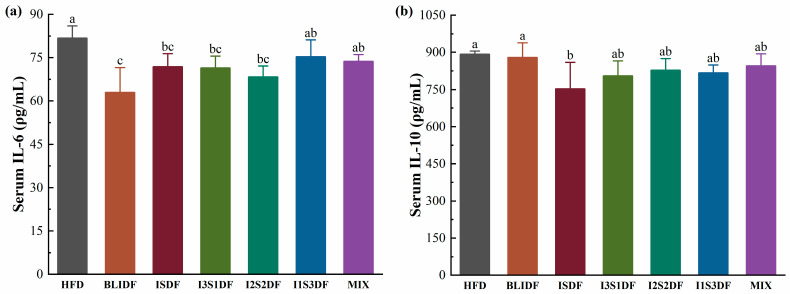
Effects of soluble and insoluble dietary fibre ratios on inflammation of obese mice. (**a**) IL-6. (**b**) IL-10. Data are presented as means ± SD (*n* = 6). Differences between each group corresponding to different letters are statistically significant (*p* < 0.05).

To evaluate the effects of soluble and insoluble dietary fibre ratios on inflammation levels and antioxidant ability in obese mice, the serum inflammatory factors IL-6 and IL-10 and the antioxidant activity-related indicators MDA and SOD were determined in our study. As shown in Figure 9a, BLIDF, ISDF, I3S1DF and I2S2DF reduced the serum IL-6 levels in the obese mice significantly, indicating their ability to reduce the body’s inflammatory response. Additionally, only ISDF decreased the IL-10 levels in the obese mice (Figure 9b). This showed that dietary fibre may reduce the inflammatory response of the body by reducing the content of inflammatory factors.

**Figure 10:**
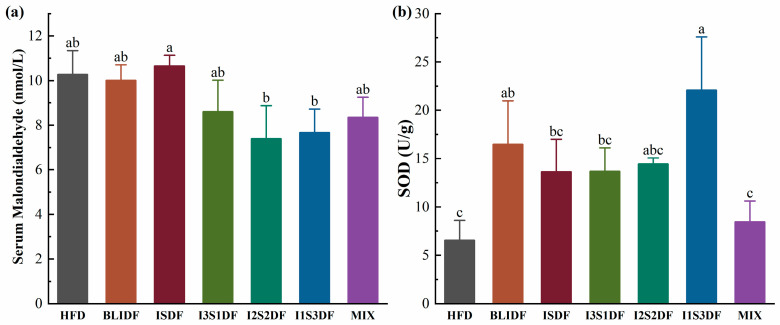
Effects of soluble and insoluble dietary fibre ratios on antioxidant ability of obese mice. (**a**) MDA. (**b**) SOD. Data are presented as means ± SD (*n* = 6). Differences between each group corresponding to different letters are statistically significant (*p* < 0.05).

As shown in Figure 10a, different dietary fibre interventions did not affect the serum MDA levels of the HFD mice, while the BLIDF and I1S3DF treatments increased the SOD activity significantly (Figure 10b). MDA serves as a biomarker that quantifies the level of lipid peroxidation within the body and thereby provides an indirect indication of the magnitude of cellular damage [24]. SOD exhibits unique physiological activity and acts as the primary antioxidant in organisms, scavenging free radicals [25]. Our results suggest that BLIDF and I1S3DF can improve the antioxidant ability of obese mice. 

### 3.7. Effects of Soluble and Insoluble Dietary Fibre Ratios on Liver Histomorphology of Obese Mice

**Figure 11:**
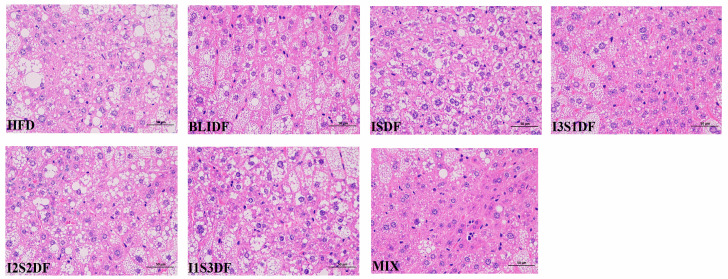
Effects of soluble and insoluble dietary fibre ratios on histopathological change in liver in obese mice (400× magnification).

The liver tissues of the mice were stained with hematoxylin and eosin (HE) and the results are shown in Figure 11. In the HFD group, the liver cytoplasm displayed rounded lipid droplets and large vacuoles, along with a disorganized arrangement of hepatic cords. All the dietary fibre interventions improved the morphological damage to the liver caused by inflammation. In particular, this condition was significantly improved in the BLIDF group after the intervention, in accordance with the decrease in serum IL-6 levels. 

### 3.8. Effects of Soluble and Insoluble Dietary Fibre Ratios on Gut Microbiota of Obese Mice

**Figure 12:**
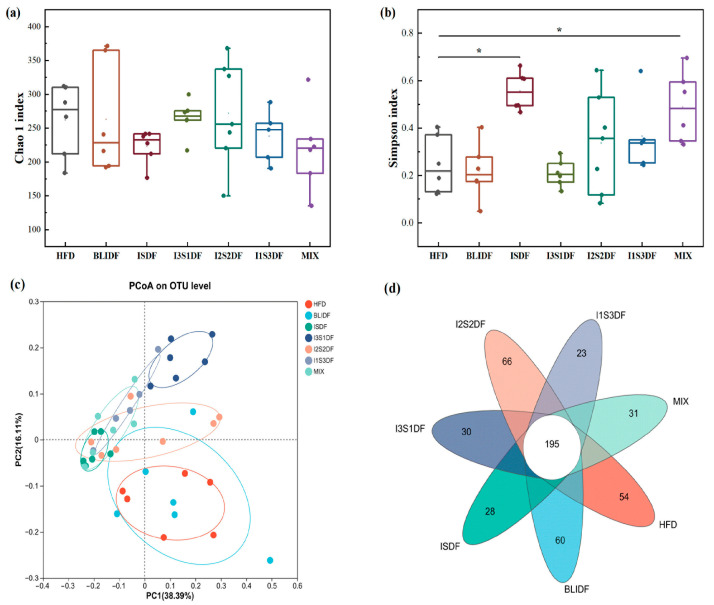
The diversity indexes of gut microbiome in mice. (**a**) Chao1 index. (**b**) Simpson index. (**c**) OTU-based PCoA analysis. (**d**) OTU-based Veen analysis. Data are presented as means ± SD (*n* = 6). * *p* < 0.05.

Dietary fibre can affect gut microbiota significantly; thus, the effects of soluble and insoluble dietary fibre ratios on gut microbiota were examined in our study. Firstly, the Chao1 index is an index utilized to estimate the total number of species within a community, whereas Simpson’s index is employed to assess species richness and the relative abundance of species within that community. As shown in Figure 12a,b, there was no significant difference between the groups in the Chao1 index, while the Simpson’s index significantly increased in the ISDF and MIX groups. It seems that ISDF and MIX may increase microbial diversity. PCoA analyses showed that all the dietary fibre intervention groups were distinctly separated from the HFD group, with only a partial overlap observed in the BLIDF group (Figure 12c). The results indicate that dietary fibre interventions can alter the composition of gut microbiota in obese mice, which is consistent with Peng’s results [5]. Specifically, the number of OTUs unique to the BLIDF and I2S2DF groups exceeded that observed in the HFD group, whereas the other intervention groups exhibited a lower abundance of specific OTUs (Figure 12d).

**Figure 13:**
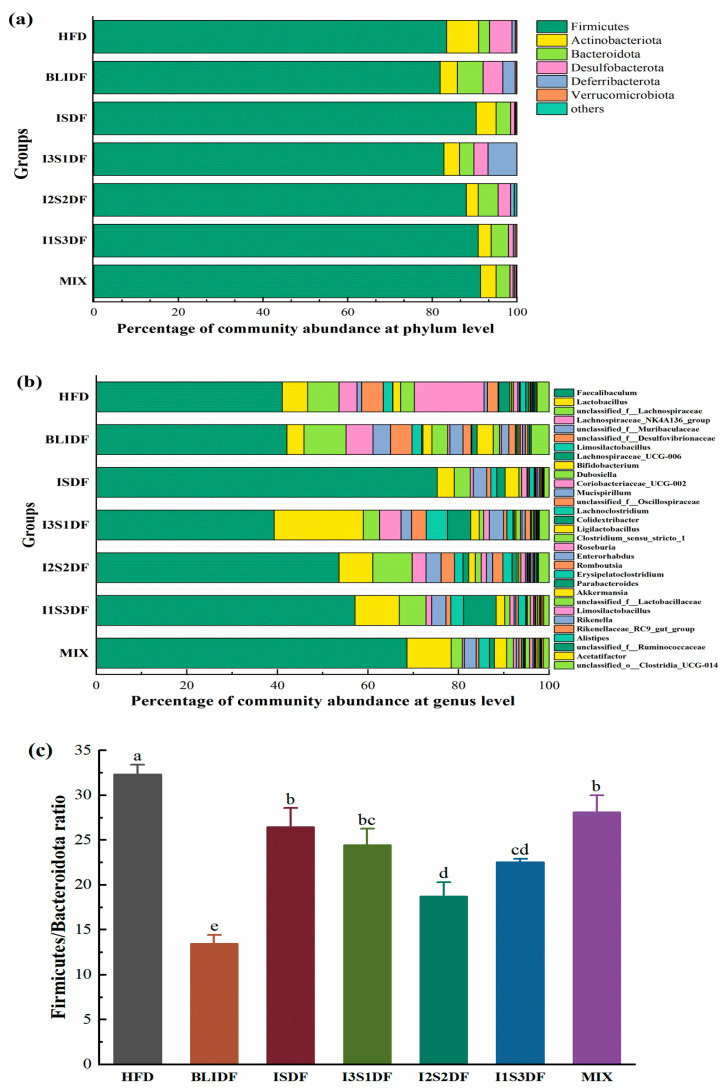
The gut microbiota composition of mice from different groups. (**a**) The gut microbiota composition at the phylum level. (**b**) The gut microbiota composition at the genus level. (**c**) The ratio of *Firmicutes* to *Bacteroidota*. Differences between each group corresponding to different letters are statistically significant (*p* < 0.05).

The gut microbiota composition of the mice in each group at the phylum and genus level is shown in Figure 13a,b. *Firmicutes* was the dominant phylum in all groups, and *Faecalibaculum* was the dominant genus in all the samples. In particular, the relative abundance of *Faecalibaculum* was much higher in the ISDF and MIX groups. It has been reported that an elevated ratio of *Firmicutes* to *Bacteroidota* (F/B) is typically associated with obesity [26]. As shown in Figure 13c, the HFD group exhibited the highest F/B ratio, and all the dietary fibre intervention groups demonstrated a significant decrease in the F/B ratio compared to the HFD group. Notably, the BLIDF group had the lowest F/B ratio, which aligned with its effect on reducing body weight. This is consistent with reported results [6], showing the correctness of our results and fully proving that the administration of soluble dietary fibre, insoluble dietary fibre or a combination of both soluble and insoluble dietary fibres can reduce obesity by affecting the gut microbiota.

**Figure 14:**
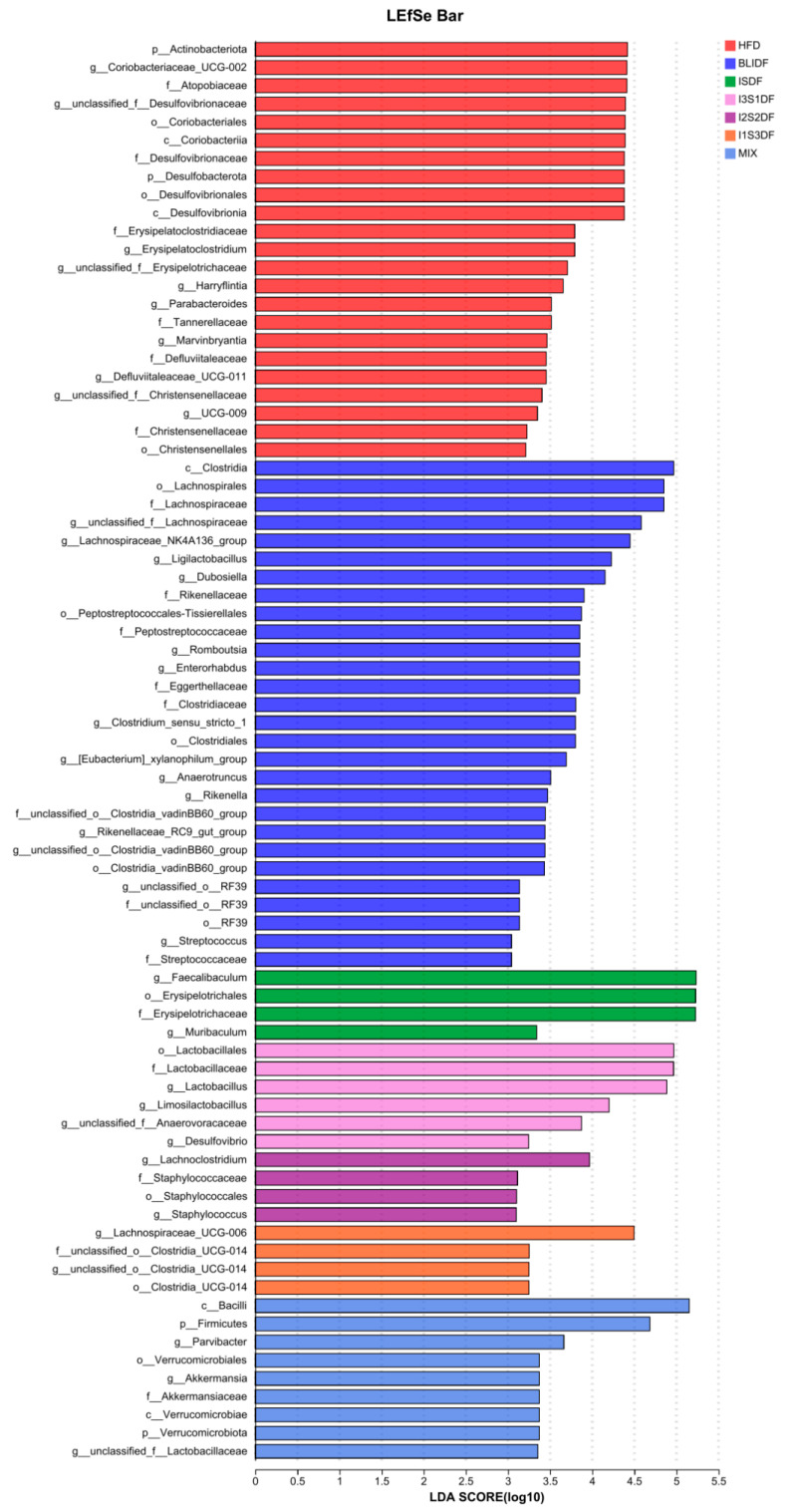
The LEfSe analysis of gut microbiota.

Further, we employed LEfSe analysis to determine the primary bacterial taxa that exhibited differential abundance among the various groups. As illustrated in Figure 14, the bacteria *p__Actinobacteriota*, *g__Coriobacteriaceae_UCG-002*, *f__Atopobiaceae*, *g__unclassified_f__Desulfovibrionaceae* and *o__Coriobacteriales* were enriched in the HFD group. The BLIDF group contained a high concentration of *c__Clostridia*, *o__Lachnospirales* and *f__Lachnospiraceae* and the ISDF group had a high abundance of *g__Faecalibaculum*, *o__Erysipelotrichales* and *f__Erysipelotrichaceae*. The bacteria *o__Lactobacillales*, *f__Lactobacillaceae* and *g__Lactobacillus* were the key microbes for the I3S1DF group, while the pivotal microbe for the I2S2DF group was *g__Lachnoclostridium*. The bacteria *g__Lachnospiraceae_UCG-006* was the dominated microbial species in the I1S3DF group. The MIX group was dominated by *c__Bacilli*, *p__Firmicutes* and *g__Parvibacter*.

*Faecalibaculum*, a significant producer of butyric acid that possesses anti-inflammatory properties [27], has been regarded as “the next generation of anti-inflammatory probiotic bacteria”. The species was predominant in the ISDF and MIX groups. This shows that the two groups may exert anti-inflammatory effects through butyrate, which is consistent with the decrease in the IL-6 content in the serum. The I3S1DF group was enriched in *Lactobacillus*. It has been demonstrated that *Lactobacillus* has several beneficial functions, such as lowering cholesterol, maintaining the balance of intestinal flora, enhancing immunity and promoting nutrient absorption [28]. This suggests that I3S1DF may alleviate the obesity of mice via *Lactobacillus*. In short, our results showed that different dietary fibre and different soluble and insoluble dietary fibre ratios could enrich distinct beneficial gut microbes.

## 4. Conclusions

In this study, we examined the effects of different ratios of soluble and insoluble dietary fibres on body weight, glycolipid metabolism and gut microbiota of obese mice. After eight weeks of dietary fibre intervention, it was found that each intervention had different effects on reducing body weight and alleviating glucose and lipid metabolism abnormalities in the mice. Among them, BLIDF significantly reduced the body weight gain and FBG level of the obese mice, and its effect on improving glucose tolerance was the most obvious. At the same time, BLIDF also significantly reduced the content of IL-6 and increased SOD activity in the serum of the obese mice, which was consistent with the liver HE staining results. This suggests that BLIDF can alleviate inflammation of the body. ISDF significantly reduced the body weight gain, FBG level and the content of IL-6 in the serum of the obese mice. I3S1DF significantly reduced the FBG level, improved the mice glucose tolerance, decreased insulin resistance and reduced the serum LDL-C, UA and IL-6 levels, indicating that I3S1DF can alleviate lipid metabolism abnormalities in obese mice. The I2S2DF group exhibited a significant effect in lowering FBG level, enhancing the glucose tolerance ability of obese mice and decreasing the serum concentration of IL-6 in mice. I1S3DF significantly reduced the FBG level, decreased insulin resistance, reduced the serum level of LDL-C and UA, and increased the SOD activity. The results suggest that it could alleviate the abnormalities of lipid metabolism and liver injury. The MIX dietary fibre intervention reduced the body weight gain and UA levels. The soluble to insoluble dietary fibre ratio of the MIX group was close to that of the I1S3DF group. However, their effects on body weight and glycolipid metabolism were different, suggesting that the source of dietary fibre plays an important role. In addition, different dietary fibre interventions increased the abundance of beneficial gut microbes differently. For example, I3S1DF significantly increased the abundance of *o__Lactobacillales*, *f__Lactobacillaceae* and *g__Lactobacillus*, and ISDF and MIX increased the abundance of *g__Faecalibaculum* and *p__Firmicutes*, respectively. Further, all the dietary fibre interventions significantly reduced the ratio of *Firmicutes* to *Bacteroidota*, suggesting that dietary fibre interventions can alleviate the intestinal flora imbalance induced by obesity. 

In conclusion, different ratios of soluble to insoluble dietary fibre can improve the abnormal glycolipid metabolism and intestinal flora imbalance of obese mice differently. Therefore, understanding the effects of different soluble to insoluble dietary fibre ratios on health will provide new strategies for effective treatments for obesity, hyperglycemia and hyperlipidemia.

## Figures and Tables

**Table 1:** The dietary fibre and other nutrient composition in barley leaf powder.

Nutrients	Content (g/100 g Barley Leaf Powder)
Protein	17.13 ± 0.494 ^a^
Fat	0.32 ± 0.163
Carbohydrate	14.98 ± 0.424
Insoluble dietary fibre	55.46 ± 0.989
Soluble dietary fibre	0.89 ± 0.989
Moisture	10.13 ± 0.382
Ash	0.75 ± 0.240

^a^ Data are expressed as mean ± SD from three independent experiments.

## Data Availability

The original contributions presented in the study are included in the article, further inquiries can be directed to the corresponding author.
